# Interventions to change maternity healthcare professionals’ behaviours to promote weight-related support for obese pregnant women: a systematic review

**DOI:** 10.1186/s13012-014-0097-9

**Published:** 2014-08-05

**Authors:** Nicola Heslehurst, Lisa Crowe, Shannon Robalino, Falko F Sniehotta, Elaine McColl, Judith Rankin

**Affiliations:** Institute of Health & Society, Newcastle University, Baddiley-Clark Building, Richardson Road, Newcastle upon Tyne, NE2 4AX England UK; Clinical Trials Unit, Newcastle University, Newcastle upon Tyne, England UK

**Keywords:** Systematic review, Healthcare professionals, Behaviour change, Obesity, Pregnancy, Weight management

## Abstract

**Background:**

There has been a rapid increase in the publication of guidelines for managing obesity and weight gain during pregnancy over the past five years. Healthcare professionals have identified multiple barriers to this area of practice, including the need to improve their communication skills, beliefs that pregnant women will have negative reactions to weight-related discussions, and a lack of weight management knowledge. This systematic review aimed to identify: the effectiveness of interventions in changing healthcare professionals’ practice relating to maternal obesity or weight management during pregnancy; and which behaviour change techniques and modes of intervention delivery have been used in interventions to date.

**Findings:**

The search strategy included searching electronic databases, trial registers, and citation searching. Inclusion criteria were intervention studies targeted towards changing healthcare professionals’ practice in relation to maternal obesity or weight management. The searches identified 3,608 studies. However, no eligible completed studies were identified. One registered Canadian randomised controlled trial was identified. The trial includes a training intervention for family physicians with the aim of supporting adherence to gestational weight gain guidelines. The trial had not yet commenced therefore no effectiveness data were available.

**Conclusions:**

The current focus of maternal obesity and weight management research is targeted towards changing pregnant women’s behaviours. These interventions do not address the multiple healthcare professionals’ barriers to maternal obesity and weight management practice. Further research is required to identify the most effective approaches to support healthcare professionals to implement maternal obesity and weight management guidelines into practice.

**Electronic supplementary material:**

The online version of this article (doi:10.1186/s13012-014-0097-9) contains supplementary material, which is available to authorized users.

## Background

Obesity in pregnancy is increasing and has significant health implications for pregnant women and their babies. These include gestational diabetes, post-partum haemorrhage, infections, stillbirth, congenital anomalies, and the long-term development of obesity and related disease in offspring [1]-[3]. Excessive gestational weight gain incurs equivalent health risks, and increases women’s susceptibility to future obesity [4],[5]. There is some evidence that gestational weight gain or risks of comorbidities can be reduced in obese women with lifestyle interventions [6]-[8]. However, interventions targeted towards pregnant women to date have had variable success, which was highlighted in a recent systematic review, primarily due to their limited quality and power [6].

Pregnancy is considered to be a critical opportunity to engage women in health promoting behaviours, such as obesity intervention [9],[10]. Since the publication of the US Institute of Medicine (IoM) gestational weight gain guidelines in 2009 [4], there has been a rapid increase in the development of guidelines for weight management during pregnancy and the care of obese pregnant women in many countries across the world [11]-[16]. Although specific guideline recommendations vary by country of origin, the behaviours targeted towards healthcare professionals (rather than pregnant women) can be broadly grouped into two categories: diagnostic and clinical intervention behaviours (*e.g.*, additional screening for diabetes, anaesthetic reviews); and communication behaviours (*e.g.*, communicating weight status and risk, and providing lifestyle and weight gain advice).

Despite the emergence of guidelines in many countries, there is an international evidence base of healthcare professionals’ multiple barriers to maternal obesity and weight management practice. A recent systematic review identified the barriers to be complex and interacting, consistent across international settings, and primarily relating to communicating with women about their weight and providing weight management advice and support [17]. Some key barriers to practice were healthcare professionals lack of knowledge (*e.g.*, an over reliance on personal experience of weight management rather than evidence-based knowledge), a belief that there would be negative consequences of intervening (*e.g.*, women would have a negative reaction to these discussions which would impair their relationship), and some environment and resource barriers (*e.g.*, a lack of supporting weight management services) [17]. However, the review also identified that healthcare professionals were motivated to overcome barriers to practice through improving their knowledge and skills [17]. Interventions targeted at supporting healthcare professionals are required to help overcome barriers to practice and to facilitate implementation of maternal obesity and weight management guidelines.

This systematic review was carried out to inform the development of an intervention to facilitate midwives’ implementation of weight management guidelines in their clinical practice (National Institute for Health Research (NIHR) funded trial: GestationaL Obesity Weight-management: Implementation of National Guidelines (GLOWING), UK Clinical Research Network (CRN) identification number: 12610). The review aimed to answer the following research questions:

1. How effective are interventions targeted towards healthcare professionals in changing their practice in respect of maternal obesity or weight management in pregnancy?
2. What behaviour change techniques have been used to date in interventions targeted towards healthcare professionals’ weight management and maternal obesity practice?
3. What modes of intervention delivery have been utilised?

## Methods

The review protocol was registered on the 29^th^ May 2013 on the NIHR PROSPERO database of systematic reviews (PROSPERO 2013:CRD42013004409, www.crd.york.ac.uk/PROSPERO/display_record.asp?ID=CRD42013004409).

### Searching and screening

Searching for eligible studies to include in the review involved three approaches: searching electronic literature databases; citation searching; and searching clinical trials registers.

### Electronic databases

The electronic databases searched included AMED (1985 – June 2013), British Nursing Index (1992 – June 2013), CINAHL (1981 – June 2013), Cochrane Central Register of Controlled Trials (1991 – June 2013), Database of Systematic Reviews (2005 – June 2013), Database of Abstracts of Reviews and Effects, 1991 – 1^st^ quarter 2013), EMBASE (1988 – June 2013), MEDLINE (1946 – June 2013), PsycINFO (1987 – June 2013), and ASSIA (1986 – June 2013). The search was adapted from a previous Cochrane review of interventions to change the behaviour of health professionals and the organisation of care to promote weight reduction in overweight and obese adults [18]. The adapted search incorporated pregnancy specific terms. Search terms were split into five sets including terms for obesity, pregnancy, behaviour change, healthcare professionals, and trials. These were combined with thesaurus terms and truncation appropriate to individual databases (see additional file 1 for an example search in MEDLINE). The search was limited to human studies published in the English language from 1990.

### Citation searching

The reference lists of two Cochrane reviews relevant to healthcare professionals’ behaviours and organisation of care for obesity treatment of patients were searched for any included studies relevant to pregnancy [18],[19]. Any studies identified through the database searching that met the inclusion criteria were also searched for relevant citations.

### Clinical trials register

Trials registers were searched for relevant trials at any stage (including those that had not yet commenced, on-going, and completed trials). Search terms included healthcare professionals, weight management, obesity, and pregnancy. The registers searched were the metaRegister of Controlled Trials (mRCT), ClinicalTrials.Gov, EU Clinical Trials Register, UK Clinical Trials Gateway (UKCTG), UK NIHR CRN Portfolio, NIHR National Research Register (NRR) Archive Search, WHO International Clinical Trials Registry Platform (ICTRP), International Standard Randomised Controlled Trial Number (ISRCTN) Register, and the Health Technology Assessments (HTA) database.

Inclusion criteria were any study design with at least one intervention and comparison group; interventions targeted towards healthcare professionals involved in the care of pregnant women; and with the aim of changing clinical practice in relation to the management of maternal obesity or weight management. For example, this could include training or education interventions, interventions to change the organisation of care, the provision of resources to support practice, etc. Studies targeted exclusively at pregnant women rather than healthcare professionals were excluded.

The Cochrane Public Health Group Data Extraction and Assessment Template [20] was intended to be used for data extraction and quality assessment by two researchers independently. This template was developed as a guide for data extraction and assessment, and to be adapted for the requirements of the review. The template adaptations required for this review included additional information on behaviour change techniques used in the interventions, and mode of delivery variables to inform analysis [21]. The assessment component of the Cochrane tool comprises of a series of questions relating to risk of bias for specific features of the study (*e.g.*, were baseline characteristics similar? Was the study adequately protected against contamination?) [22]. The tool requires the reviewers’ to make a judgement of whether the included study has ‘low risk’ , ‘high risk, or ‘unclear risk’ of bias.

### Data analysis

A meta-analysis of the results reported in the included studies on effectiveness of the interventions was planned. A narrative summary of behaviour change techniques and modes of delivery used in interventions targeted towards healthcare professionals was planned.

## Results

**Figure 1:**
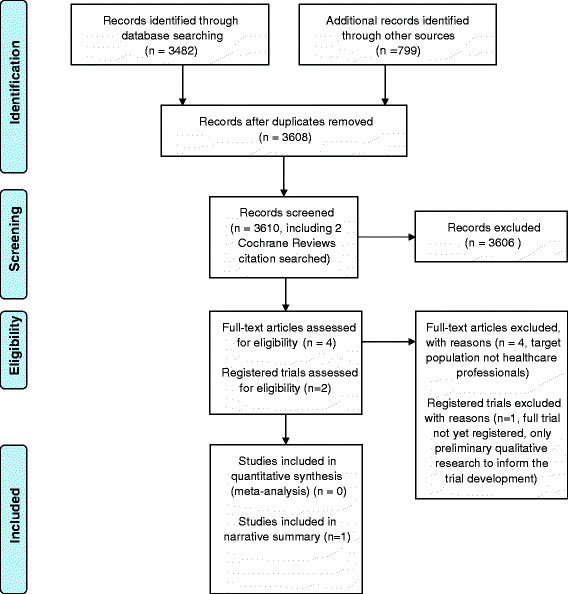
**PRISMA flow diagram.** From: Moher D, Liberati A, Tetzlaff J, Altman DG, The PRISMA Group (2009). Preferred Reporting Items for Systematic Reviews and Meta-Analyses: The PRISMA Statement. PLoS Med 6(6): e1000097. doi:10.1371/journal.pmed1000097.

The database searches identified 3,482 studies. A further 766 studies were identified through searching the trials registers. Following de-duplication, 3,608 studies remained. Additionally, two existing relevant systematic reviews for adult populations were citation searched for any pregnancy-related studies [18],[19] (Figure 1). Four published studies identified in the database search were retrieved for full review, as it was not possible to determine if they met the inclusion criteria from the title and abstract alone. However, following full paper review, these studies did not meet the inclusion criteria, as all were interventions targeted towards pregnant women rather than healthcare professionals. No potentially eligible studies were identified through citation searching.

One relevant clinical trial was identified through searching the trials registers (Table 1). The trial aims to determine efficacy of a training intervention targeted towards family physicians and the adherence of their patients to the IoM gestational weight gain guidelines. Family physicians will be randomly assigned to receive the intervention or act as controls, and the primary outcome measure is the weight gain of their patients at 38 weeks gestation (classified according to the pre-pregnancy body mass index) [23]. The trial is not yet complete and therefore does not provide data on the effectiveness of the intervention. No further details of the behaviour change techniques and mode of delivery employed in this study were provided. No further eligible studies were identified through the searches.

**Table 1:** **Characteristics of included studies**

Trial name	Trial register	Trial location	Target population	Research question	Trial design	Stage of trial
Management of gestational weight gain by family physicians: seeking congruence with guidelines	ClinicalTrials.gov NCT01803698	Canada	Family Physicians	What impact does training family physicians to regularly refer to the IoM trajectories and provide feedback about GWG (‘training in the use of IOM charts’) during routine prenatal visits, compared to usual care, have on congruence of total GWG with IoM guidelines?	Randomised Controlled Trial	Not yet open for recruitment. Planned timescale January 2014 to August 2015.

IoM: Institute of Medicine; GWG: gestational weight gain.

## Conclusions

Throughout the screening process, all intervention-based studies identified which were relevant to maternal obesity or weight management were targeted towards pregnant women rather than healthcare professionals, with the exception of one registered clinical trial, yet to recruit. There are existing systematic reviews of the effectiveness of interventions at managing gestational weight gain or reducing obesity-related risks during pregnancy [6]-[8] but all of these are focused on changing the behaviour of the pregnant woman rather than the health care professionals involved in her care. The evidence base to date is unclear on the best approach to change women’s weight-related behaviours during pregnancy due to the limited success of existing interventions. There is some evidence that dietary interventions are more successful than physical activity interventions at reducing weight-related obstetric risk, but there is a lack of good quality, and adequately powered, studies to determine this [6]. The limited effectiveness of interventions targeted towards pregnant women’s behaviours is consistent in international studies, as are the barriers to practice identified by healthcare professionals. This suggests that these factors are not overly culturally or context-specific to the country of origin. A key reason for the limited success of interventions to date may be that those targeted towards pregnant women do not address the multiple barriers to practice that healthcare professional’s face. Good quality process evaluations of future interventions should explore the potential influence of healthcare professionals’ barriers to practice on the effectiveness of the intervention.

This systematic review was not able to answer our research questions. Publication of empty reviews is an important practice. A Cochrane Empty Reviews Project was established in 2010 to develop reporting guidelines for empty reviews [24]. One important aspect of publishing empty reviews is to identify the gaps in the evidence-base, and stimulate primary research to increase the availability of evidence for future review updates [25]. This systematic review has identified a lack of published and ongoing research to support healthcare professionals to overcome their barriers to practice. It is clear that interventions are urgently required to facilitate the implementation of international maternal obesity and weight management guidelines.

## Additional file

## Electronic supplementary material

Additional file 1: Electronic Database Search for “Interventions to change maternity healthcare professionals’ behaviours to promote weight-related support for obese pregnant women: a systematic review”.(DOCX 21 KB)

Below are the links to the authors’ original submitted files for images.

Authors’ original file for figure 1
