# Supplementary material for: Interventions to change maternity healthcare professionals’ behaviours to promote weight-related support for obese pregnant women: a systematic review
Source: Implement Sci. 2014 Aug 5;9:97. doi: 10.1186/s13012-014-0097-9 (PMC4244067; doi:10.1186/s13012-014-0097-9)
Supplement: Supplementary file 1 — Additional file 1: Electronic Database Search for “Interventions to change maternity healthcare professionals’ behaviours to promote weight-related support for obese pregnant women: a systematic review”.(DOCX 21 KB) [file 13012_2014_97_MOESM1_ESM.docx]

**Additional File 1: Electronic Database Search for “Interventions to change maternity healthcare professionals' behaviours to promote weight-related support for obese pregnant women: a systematic review”**

PIOS

Population: *Healthcare Professionals involved in the care of pregnant women*

Intervention: *Behaviour (clinical practice) change interventions*

Outcome: *Maternal obesity and weight management practice*

Study Design: *Interventions with at least one comparator group*

Limits: *Human trials, since 1990, published in English*

Example Medline search

1. exp overweight/

2. exp obesity/

3. (obes$ or overweight$).tw.

4. Weight loss/

5. *body mass index/

6. *weight gain/

7. (bmi or body mass index).ab,ti.

8. (body size or bodysize or body-size).ab,ti.

9. (fat or fatness).ab,ti.

10. ((maternal or mother*) adj bod*).ab,ti.

11. obes*.ab,ti.

12. (Overweight or over-weight).ab,ti.

13. (weight adj (gain or maintenance or management)).ab,ti.

14. (body adj weight).ab,ti.

15. or/1-14

16. exp pregnancy/

17. *pregnant women/

18. Fetlility/

19. exp fertilization in vitro/

20. exp delivery, obstetric/

21. (antenatal* or ante-natal*).tw.

22. (anteoartum or ante-partum).tw.

23. birth.tw.

24. Conception.tw.

25. fertile*.tw.

26. Gestation.tw.

27. gravid*.tw.

28. infertile*.tw.

29. (interpregnancy or interpregnancy).tw.

30. (intrapartum or intra-partum).tw.

31. (intra-pregnancy or intrapregnancy).tw.

32. (IVF or In vitro fertilisation or in-vitro-fertilisation or In vitro fertlilization or In-vitro fertilization).tw.

33. (labor or labour).tw.

34. maternal.tw.

35. maternity.tw.

36. natal*.tw.

37. (parity or parous).tw.

38. (periatal* or peri-natal*).tw.

39. (postnatal* or post-natal*).tw.

40. (postpartum or post-partum).tw.

41. (preconception or pre-conception).tw.

42. pregnan*.tw.

43. (prenatal* or pre-natal*).tw.

44. (prepregnancy or pre-pregnancy).tw.

45. reproducti*.tw.

46. (subfertil* or sub-fertil*).tw.

47. (nulligravid or nulli-gravid).tw.

48. (multigravid or multi-gravid).tw.

49. (primigravid or primi-gravid).tw.

50. or/16-49

51. 15 and 50

52. exp education, continuing/

53. (education$ adj2 (program$ or intervention? or meeting? or session? or strategy$ or workshop? or visit?)).tw.

54. (behavio?r$ adj2 intervention?).tw.

55. *pamphlets/ or (leaflet? or booklet? or poster or posters).tw.

56. ((written or printed or oral) adj information).tw.

57. (information$ adj2 campaign).tw.

58. (education$ adj1 (method? or material?)).tw.

59. Outreach.tw.

60. ((opinion or education$ or influential) adj1 leader?).tw.

61. Facilitator?.tw.

62. Academic detailing.tw.

63. Consensus conference?.tw.

64. Practice guidelines as topic/

65. *guideline adherence/

66. Practice guideline?.tw.

67. (guideline? adj2 (introduce$ or issu$ or impact or effect? or disseminat$ or distribut$)).tw.

68. ((effect? or impact or evaluat$ or introduce$ or compar$) adj2 training program$).tw.

69. *reminder systems/

70. Reminder?.tw.

71. (recall adj2 system$).tw.

72. (prompter? or prompting).tw.

73. Algorithm?.tw.

74. *feedback/ or feedback.tw.

75. (feedback adj1 (loop? or control? or regula$ or mechanism? or inhib$ or system? or circuit? or sensory or visual or audio$ or auditory)).tw.

76. Chart review$.tw.

77. ((effect? or impact or records or chart?) adj2 audit).tw.

78. Compliance.tw.

79. Marketing.tw.

80. exp *Reimbursement Mechanisms/

81. fee for service.tw.

82. *capitation fee/

83. *deductibles/ and coinsurance/

84. Cost shar$.tw.

85. (copayment? or co payment?).tw.

86. (prepay$ or prepaid or prospective payment?).tw.

87. *hospital charges/

88. Formula$.tw.

89. Fundhold$.tw.

90. *Medicaid/

91. Medicare/

92. Blue cross.tw.

93. Conselling.tw.

94. Development.tw.

95. guideline implementation.tw.

96. behaviour change.tw.

97. guide for practitioners.tw.

98. guide for health?care professionals.tw.

99. clinical behaviour.tw.

100. Behavio?r.tw.

101. Practice$.tw.

102. Community health planning/

103. Community health systems/

104. Delivery of healthcare/

105. Health care systems/

106. Health information systems/

107. Health systems agencies/

108. Commissioning.tw.

109. or/52-108

110. 51 and 109

111. exp Maternal Health Services/

112. *Family Planning Services/

113. exp perinatal care/

114. *preconception care/

115. *prenatal care/

116. *midwifery/

117. *Birthing Centers/

118. *Hospitals, Maternity/

119. exp health personnel/

120. ((healthcare adj professional*) or health-care professional* or health care professional* or HCP).ab,ti.

121. (primary adj care).ab,ti.

122. (community adj services).ab,ti.

123. (nurse or nurses).ab,ti.

124. (maternity adj unit*).ab,ti.

125. health professional*.ab,ti.

126. (general practitioner* or GP).ab,ti.

127. pediatric*.ab,ti.

128. (midwife* or midwive*).ab,ti.

129. obstetri*.ab,ti.

130. doctor*.ab,ti.

131. ((birth or birthing) adj cent*).ab,ti.

132. (family adj1 planning).ab,ti.

133. Dietician*.ab,ti.

134. Nutritionist*.ab,ti.

135. gyn?ecolog*.ab,ti.

136. OBGYN.ab,ti.

137. (health adj visitor*).ab,ti.

138. (secondary adj care).ab,ti.

139. (multi-disciplinary adj (team or care)).ab,ti.

140. (maternity adj service*).ab,ti.

141. (maternity adj ward*).ab,ti.

142. practioner.ab,ti.

143. (care-provider or care provider or careprovider).ab,ti.

144. exp Health Personnel/

145. clinical pharmacist?.tw.

146. paramedic?.tw.

147. nutritionist?.tw.

148. dieti#ian?.tw.

149. *patient care team/

150. exp patient care planning/

151. (team? adj2 (care or treatment or assessment or consultation)).tw.

152. (integrat$ adj2 (care or service?)).tw.

153. (care adj2 (coordinat$ or program$ or continuity)).tw.

154. (case adj1 management).tw.

155. *ambulatory care/

156. *home care services/

157. *hospices/

158. *office visits/

159. *house calls/

160. *day care/

161. *aftercare/

162. *community health nursing/

163. (chang$ adj1 location?).tw.

164. (domicillary or domiciliary).tw.

165. (home adj1 treat$).tw.

166. day surgery.tw.

167. *health facilities/ or *academic medical centers/ or *ambulatory care facilities/ or *birthing centers/ or *health facilities/

168. proprietary/ or *hospital units/ or exp hospitals/ or *pharmacies/ or *physicians offices/ or *rehabilitation centers/ or exp *residential/

169. facilities/

170. *group practice/ or *institutional practice/ or *nursing faculty practice/ or *partnership practice/ or *private practice/

171. exp *Neonatology/

172. or/111-171

173. 110 and 172

174. randomized controlled trial.pt.

175. Randomized Controlled Trials as Topic/

176. random allocation/

177. double blind method/

178. single blind method/

179. clinical trial.pt.

180. exp Clinical Trial/

181. (clin$ adj25 trial$).tw.

182. ((singl$ or doubl$ or trebl$ or tripl$) adj25 (blind$ or mask$)).tw.

183. placebo/

184. placebo$.tw.

185. random$.tw.

186. research design/

187. comparative study/

188. exp evaluation studies/

189. follow up studies/

190. prospective studies/

191. (control$ or prospective$ or volunteer$).ti,ab.

192. intervention$.ti,ab.

193. or/174-192

194. animal/

195. human/

196. 193 and 195

197. 173 and 196

198. limit 197 to (english language and humans and yr=1990-Current)

199. 15 and 109 and 173 and 193

200. limit 199 to (english language and humans)

201. 15 and 50 and 172 and 193

202. limit 201 to (english language and humans)

203. limit 202 to yr=1990-Current

204. 203 not 198

205. 199 not 198

206. limit 205 to (english language and humans)
