# Supplementary figures and images for: Interventions to change maternity healthcare professionals’ behaviours to promote weight-related support for obese pregnant women: a systematic review
Source: Implement Sci. 2014 Aug 5;9:97. doi: 10.1186/s13012-014-0097-9 (PMC4244067; doi:10.1186/s13012-014-0097-9)

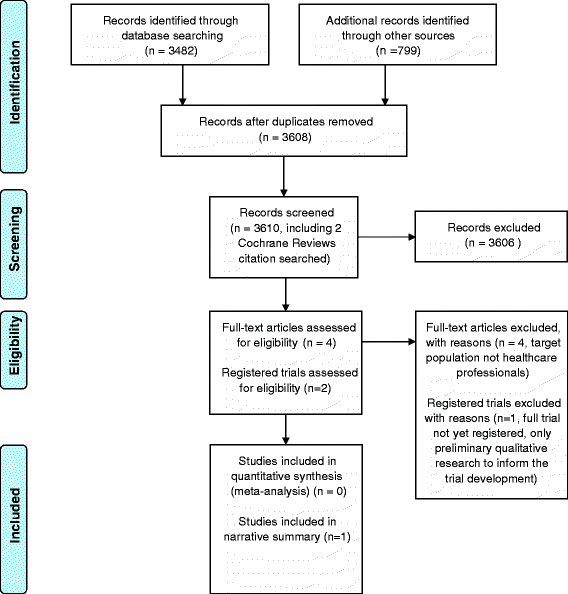

Supplement: Supplementary file 2 — Authors’ original file for figure 1 [file 13012_2014_97_MOESM2_ESM.gif]
